# Patterns of Genetic Diversity and Gene Flow Associated With an Aridity Gradient in Populations of Common Mole-rats, *Cryptomys hottentotus hottentotus*

**DOI:** 10.1093/gbe/evae144

**Published:** 2024-07-02

**Authors:** Hana N Merchant, Anastasia Ivanova, Daniel W Hart, Cristina García, Nigel C Bennett, Steven J Portugal, Chris G Faulkes

**Affiliations:** Department of Biological Sciences, School of Life and Environmental Sciences, Royal Holloway University of London, Egham, Surrey TW20 0EX, UK; School of Biological and Behavioural Sciences, Queen Mary University of London, London, UK; School of Biological and Behavioural Sciences, Queen Mary University of London, London, UK; Department of Zoology and Entomology, University of Pretoria, Pretoria, Gauteng, South Africa; Department of Biological Sciences, School of Life and Environmental Sciences, Royal Holloway University of London, Egham, Surrey TW20 0EX, UK; Mammal Research Institute, Department of Zoology and Entomology, University of Pretoria, Pretoria, Gauteng, South Africa; Department of Biological Sciences, School of Life and Environmental Sciences, Royal Holloway University of London, Egham, Surrey TW20 0EX, UK; Department of Biology, University of Oxford, Oxford OX1 3SZ, UK; School of Biological and Behavioural Sciences, Queen Mary University of London, London, UK

**Keywords:** arid-dwelling mammals, Bathyergidae, environmental adaptation, genetic adaptation, genome-wide variation, landscape genomics

## Abstract

Genetic adaptation is the change of a population toward a phenotype that best fits the present ecological conditions of the environment it inhabits. As environmental conditions change, allele frequencies shift, resulting in different populations of the same species possessing genetic variation and divergent phenotypes. Cooperatively breeding common mole-rats (*Cryptomys hottentotus hottentotus*) inhabit environments along an aridity gradient in South Africa, which provides an opportunity for local genetic adaptations to occur. Using one mitochondrial gene (cytochrome b) and 3,540 SNP loci across the whole genome, we determined the phylogenetic relationship, population structure and genetic diversity of five populations of *C. h. hottentotus* located along an aridity gradient. Mitochondrial data identified population-specific clades that were less distinct in the two mesic populations, potentially indicating historical or recent gene flow, or the retention of ancestral haplotypes. Arid and semi-arid populations formed a distinct cluster from the non-arid populations. Genetic diversity and gene flow were higher in arid-dwelling individuals, suggesting greater connectivity and interactions between colonies in arid regions in comparison to mesic ones. Using an Aridity Index, we determined that isolation by environment, rather than isolation by geographical distance, best explains the genetic distance between the populations. Further analyses using target loci may determine if there are differing underlying genetic adaptations among populations of *C. h. hottentotus*. These analyses could help unravel population differences in response to environmental factors within a subspecies of bathyergid mole-rat and determine the adaptive capacity of this small nonmigratory subterranean rodent species in response to aridification in the face of climate change.

**Significance**

This study identifies population-level genomic differences that occur in association with extreme arid conditions and highlights that some populations may be more at risk in response to climate change and aridification than others as a result of changes in allelic composition and genetic diversity. The potential for loss of distributional range in this species could thus be high, and therefore further investigations of their genetics and specific adaptations may influence conservation policies to ensure the survival of this and related species.

## Introduction

Genetic adaptation is affected by population-level genetic processes such as genetic drift, natural selection, random mutations, and gene flow (Cortázar-Chinarro et al. 2017). The variation in selective pressures between habitats influences the occurrences and effects of these processes resulting in genetic variation (McCallum et al. 2014) and divergent phenotypes (Orr 2005; Hoffmann and Willi 2008). Certain environments require extreme and more specialized adaptations, and thus can exert a stronger influence on the genetic differentiation of populations than actual geographical (physical) distance (Wang and Bradburd 2014); often the more extreme the environment, the greater the adaptive drive. One example of an extreme environment is xeric biomes, which include deserts and arid regions (Harris et al. 1998). Arid environments are typically characterized by extreme water scarcity, high temperatures, and minimal vegetation, all of which present challenges that influence the adaptive strategies of the organisms living in these conditions (Harris et al. 1998).

Certain genomic regions interface more with the environment, and thus will be more relevant regarding adaptation to extreme desert conditions (Rocha et al. 2021). Previously identified genes linked to mammalian desert adaptations include those associated with energy storage, low energy expenditure, adaptive tolerance to starvation, water retention, stress responses (such as oxidative damage and heat stress), radiation tolerance, and metabolism of toxins from the diet (Rocha et al. 2021). The use of genome-wide single nucleotide polymorphisms (SNPs) has successfully located these regions within the genome that may be associated with environmental stressors, specifically those associated with arid environments (Rocha et al. 2021). Further analyses using genome-wide studies may be used to examine population structure and genetic variability. Landscape genetic analysis can shed light on intraspecific genetic variability, and the adaptive capacity of various populations. Such approaches are becoming particularly critical in light of global rapidly changing environments, and provide insight into population connectivity, habitat structure, dispersal patterns, and biodiversity (Drake et al. 2022). For example, desert-dwelling cactus mice (*Peromyscus eremicus*) are a prime example of a species known to display heat tolerance and dehydration adaptations. Using whole genome data, Tigano et al. (2020) identified sites across the genome associated with selective sweeps. Functional annotations of candidate genes revealed signatures of selection at sites with genes related to cellular mechanisms used to cope with thermal and hyperosmotic stress. Additionally, population structure analyses identified distinct clusters of populations isolated by geographical barriers, regardless of geographic proximity, driven by the limited dispersal ability of *P. eremicus* (Tigano et al. 2020). Moreover, a study on greater gliders (*Petauroides volans*) different geographic locations identified candidate markers based on genome-wide SNPs. Geographically isolated populations showed low genetic diversity, and candidate markers indicated the detection of regions of adaptation to temperature using genotype–environment association (Knipler et al. 2023). Such explorations enable a deeper understanding of population genetics, coupled with local adaptations in response to an aridity gradient. These studies highlight the impact of environmental and geographical features on genetic diversity and variation. Nevo and Beiles (1989) compared genetic diversity in species of crickets, land snails, lizards, mice, and pocket gophers that inhabit the desert, Mediterranean, and steppe regions of Israel. This study found that on Mount Carmel, also termed “the Evolution Canyon”, genetic diversity and heterozygosity increased toward ecologically heterogeneous environments. This study also found evidence of increased genetic diversity in Mediterranean species thriving in and near the harsher desert environment (Nevo and Beiles 1989), whilst genetic diversity was low in the species existing solely in the arid habitat. The result from this study necessitates further investigation into the population genetics and structure of such species whose range spans different biomes, from arid to mesic, to further understand how aridity influences genetic diversity. A deeper understanding of intraspecific genetic variation has numerous practical applications. Genetic diversity, for example, can be a reliable indicator of translocation success in reintroduced individuals (Scott et al. 2020). Scott et al. (2020) found that Mojave Desert tortoises (*Gopherus agassizii*) with higher individual heterozygosity survived at much higher rates than those with lower individual heterozygosity when reintroduced into wild populations.

African mole-rats (Bathyergidae) are subterranean rodents found across sub-Saharan Africa (Faulkes and Bennett 2021). Mole-rats are an ideal clade for comparative analyses because they occupy a broad distribution crossing many climatic regions, coupled with variations in food availability and soil type. The ability of common mole-rats and other African mole-rat species to persist and thrive in arid regions of Africa is due to the adaptive benefits of group living which increases the energy allocations dedicated to foraging and locating stochastically distributed food sources (Jarvis et al. 1994). The aridity food distribution hypothesis (AFDH) posits that larger numbers of individuals per colony are found in arid regions to increase the chances of finding stochastically distributed food resources to secure sufficient energy allocations of the colony dedicated to foraging (Jarvis et al. 1994). The scarcity of food is thought to limit the number of individuals that can exist in these arid environments, and thus reduce genetic diversity and connectivity between colonies in arid populations (Visser et al. 2018). Two of the three social genera, *Fukomys* and *Cryptomys*, share common ancestry around 10 to 12 million years ago. Throughout much of the range of *Fukomys*, speciation and diversity have been shaped by landscape evolution driven by physical, ecological, and climatic changes linked to the formation of the African Rift Valley. Cladogenesis is associated with significant bouts of volcanic activity (Faulkes et al. 2010, 2017), whilst changes in drainage patterns within the major river systems of the Zambezian region in South-Central Africa are particularly important for structuring present populations and potential speciation events in some *Fukomys* populations (Van Daele et al. 2004, 2007, 2013). In comparison, the genus *Cryptomys* has been restricted to the less geologically active southern African region, diversifying almost entirely across South Africa (Faulkes et al. 2017). Within the genus, common mole-rats (*Cryptomys hottentotus hottentotus*) are unusual in being found along an environmental cline in South Africa, ranging from mesic to arid habitats. This allows for within-species comparison, between different populations in widely different environments. Population-level differences in morphology have recently been identified in response to an aridity gradient (Merchant et al. 2024), thus indicating that aridity is acting as a selective pressure on populations of *C. h. hottentotus*. Further exploration into genetic adaptation in response to aridity is required to determine the level of adaptation occurring at the population level.

Using microsatellite genetic markers, Bishop et al. (2004, 2007) explored, for the first time, *C. h. hottentotus* colony structure and relatedness in both arid and mesic populations, revealing that colonies are dominated by outbreeding family groups consisting of a breeding queen and siblings with sometimes non-related adult individuals. Paternity was found to be primarily attributed to the dominant male in the colony; however, the presence of extra-colony paternity by floating males was shown at both sites by the occurrence of offspring whose paternity could not be assigned to the resident breeding male. Although the study sites differed considerably in area, the results suggested that movement between colonies by individual mole-rats was greater at the mesic (Somerset West) site (Bishop et al. 2004, 2007).

In this study, we use targeted genome-wide SNPs, baited to target ultra-conserved elements (UCEs). UCEs are highly conserved regions of the genome, and 481 UCE sites are shared among extremely divergent taxa (Bejerano et al. 2004). The flanking DNA regions of UCEs are highly variable, and the SNP data from these variable regions are useful for reconstructing population-level evolutionary histories (Smith et al. 2014). The role of UCEs is currently not fully known, however, they have been associated with gene regulation and organismal development (Sandelin et al. 2004; Pennachhio et al. 2006). UCEs are believed to be functional and important parts of the genome and are almost universally conserved across evolutionary distant taxa. Gene knockouts of UCE loci in mice produced viable, fertile offspring however, and thus, their role remains cryptic (Ahituv et al. 2007).

We tested hypotheses to infer the evolutionary processes and ecological drivers that underpin genetic variation across the distribution of *C. h. hottentotus* and determine population differences in genetic diversity and gene flow. Using mitochondrial DNA (cytochrome b) and genome-wide SNP data for 39 individuals from 29 colonies across five *C. h. hottentotus* populations varying in Aridity Index (two arid, one semi-arid, and two non-arid), we determined if genetic diversity is associated with aridity, and identified differences in population structure and movement patterns in response to aridity. We predicted that the evolutionary relationships of the different populations would indicate an arid–mesic split. We predicted that genetic differentiation would be linked to aridity, and that genetic diversity and gene flow would be higher in non-arid populations due to the greater prevalence of food resources, and thus a larger effective population size in the environment. Arid populations were predicted to be under stronger selection pressures due to the harsh environmental conditions and consequently have lower genetic diversity and increased directional selection for genes associated with arid adaption. Additionally, we predicted that the genetic structure of the populations would reveal separate clusters for the arid and non-arid populations and genetic distance was related to biome type (arid, semi-arid, and non-arid/mesic).

## Results

### Evolutionary Relationships and Mitochondrial Variation Along an Aridity Gradient

*Cryptomys h. hottentotus* mtDNA-cytochrome b gene (1,140 bp) sequences were obtained for 39 individuals representing five populations. The mitochondrial cytochrome b data demonstrated 69 sites recorded with SNPs. Overall mtDNA-cytochrome b nucleotide diversity (π) across all populations was low (0.014). Within the biomes, π was 0.0064 for arid populations, 0.0022 for semi-arid, and 0.0042 for mesic populations. The number of haplotypes was the highest in arid areas (9), and the lowest in semi-arid (5). Tajima's *D* statistic returned a negative value of −0.22, indicating a higher-than-expected number of low-frequency polymorphisms.

In total, 20 haplotypes were identified, and 19 out of 20 haplotypes (95%) were private (i.e. restricted to that population). These include all haplotypes from Somerset West, Darling, and Klawer. One haplotype (supplementary table S1, Supplementary Material online, Haplotype 1) was present in two different populations, Steinkopf and No Heep. Non-synonymous mutations which lead to a change in the protein sequence were found in 9 out of 20 haplotypes.

The hierarchical (nested) analysis of molecular variance (AMOVA) for cytochrome b (Table 1) revealed very low genetic variation (3.3%) and the highest fixation index (ΦST = 0.97) within populations. High genetic variation (32.7%) and a high fixation index (ΦSC = 0.91) were found between populations. Both these results were statistically significant (*P* < 0.001). The highest differences (64.0%) and the lowest fixation index (ΦCT = 0.64) are found among the biome groups; however, this result was not statistically significant (*P* = 0.07).

**Table 1:** Nested analysis of molecular variance (AMOVA) among mtDNA-cytochrome b sequences across six populations of *C. h. hottentotus*

Source of variation	Degrees of freedom	Variation (%)	Fixation indices	*P* value
Among biomes	2	64.0	0.63984	=0.066^NS^
Among populations	3	32.7	0.90742	<0.001*
Within populations	30	3.3	0.96666	<0.001*

Biomes are defined by the aridity categories based on the Aridity Index for each population (arid, semi-arid, or mesic). Significant results are in bold and marked with an asterisk (*). Nonsignificant results are denoted by NS.

**Fig. 1.**
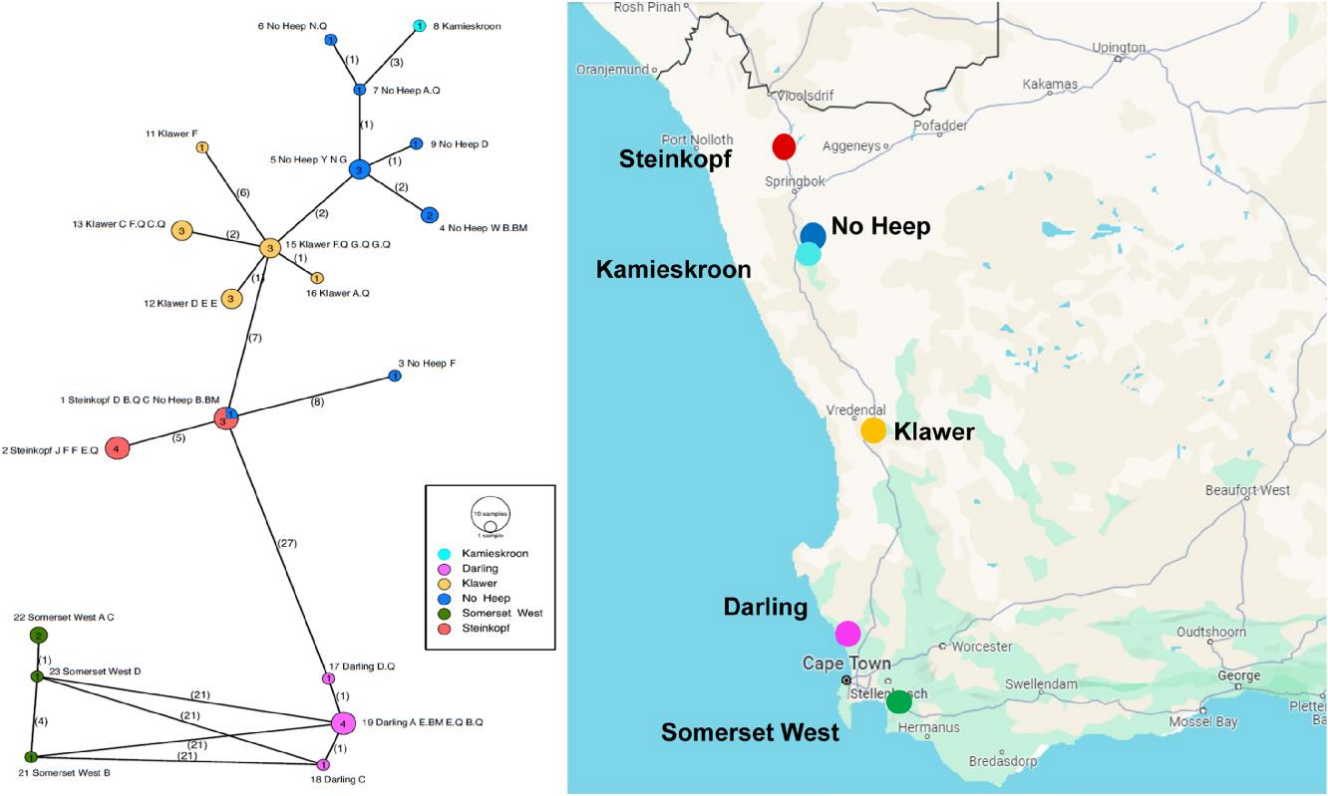
Minimum Spanning Network for six populations of *C. h. hottentotus* haplotypes. Circles represent haplotype, and size and numbers within the circles indicate the number of individuals that exhibit each respective haplotype. Populations are color-coded. Branch numbers indicate the number of mutations between each haplotype. Branch length is non-informative, and the network is unrooted. Geographical distribution of the six populations of *C. h. hottentotus* is presented on a map of South Africa. See supplementary table S1, Supplementary Material online for haplotype naming system.

The Unrooted Minimum Spanning Network (Fig. 1) identified 20 cytochrome b haplotypes overall. Haplotypes that belong to individuals from the same populations clustered together and exhibited a lower degree of genetic differentiation from each other (most often one to two mutations), as indicated by numbers in parentheses along the branches. Similarly, the differentiation between arid-dwelling populations was lower compared to the mesic populations. This finding is further supported by the maximum parsimony and maximum likelihood trees of the observed haplotypes (supplementary fig. S1a and b, Supplementary Material online). The private nature of all haplotypes except for Haplotype 1 is evident, given the geographical clustering by population (Fig. 1).

### Genetic Diversity and Structure Patterns Among Populations as a Function of an Aridity Gradient

Of the genome-wide UCE data identified, 3,540 diploid nuclear loci for 39 individuals across five populations were found. In total, four out of five populations (all except Klawer) showed significant deviations from Hardy–Weinberg equilibrium (HWE) using a Chi-Squared test, and all five populations showed significant deviation from HWE using a MC permutation test (Table 2). The total fraction of locus pairs with positive *r*^2^_D_ across populations and linked loci was 0.54, which was higher than expected under random association. Deviations from linkage equilibrium were significant for all populations (values higher than 0), and linkage disequilibrium was highest for the mesic population, Darling (Ia = 265.375, *r*^2^_D_ = 0.238, *P* < 0.05), and lowest in the arid population, No Heep (Ia = 64.207, *r*^2^_D_ = 0.03, *P* < 0.05). For all populations, the unbiased heterozygosity (uH_e_) ranged from 0.147 to 0.237. The inbreeding coefficient (*F*_is_) values were above zero in Klawer and Darling, indicating that these populations have lower individual variation, and thus a deficiency of heterozygotes. Rarefied allele richness (*A*_r_) varied from 1.104 to 1.209, and the number of private alleles ranged from 25 to 283. All results are summarized in Table 2. The models identified that genetic diversity measured as *A*_r_ and heterozygosity decreased significantly with an increase in Aridity Index (AI) (supplementary fig. S2, Supplementary Material online), and the number of private alleles exhibited a quadratic relationship with AI, revealing a higher number of private alleles at extreme aridity.

**Table 2:** Genetic diversity results for five populations of *C. h. hottentotus*

Population	HWE		LD	Heterozygosity			*A*_r_	Private alleles	*F*_is_
	Chi-Squared	MCMC	*r* ^2^_D_	*H*_o_	*H*_e_	uH_e_			
Steinkopf	0.029	0.008	0.136	0.215	0.197	0.21	1.191	25	−0.082
No Heep	0.03	0.014	0.03	0.215	0.21	0.221	1.205	132	−0.018
Klawer	0.092	0.011	0.122	0.186	0.22	0.237	1.209	109	0.148
Darling	0.028	0.01	0.238	0.129	0.137	0.147	1.13	57	0.037
Somerset West	0.028	0.012	0.074	0.121	0.107	0.115	1.104	283	−0.121

For each population, the values are listed for Hardy–Weinberg equilibrium (HWE) significance results for a Chi-Squared test, and MCMC permutation test, linkage disequilibrium (LD) *r*^r^_D_ values, observed heterozygosity (*H*_o_), expected heterozygosity (*H*_e_), unbiased expected heterozygosity (uH_e_), allelic richness (*A*_r_), number of private alleles, inbreeding coefficient (*F*_is_), and number of individuals per population (n).

### Aridity as a Driver Underpinning Genetic Differentiation Patterns

**Fig. 2.**
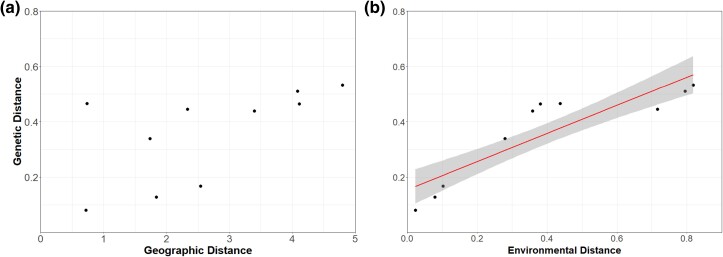
Scatter plot for a) isolation by distance shows a relationship between the genetic (*F*_st_) and geographic distances of the population pairs (*P* = 0.048), and b) isolation by environment shows a significant and linear relationship between the genetic (*F*_st_) and environmental distance (Aridity Index) of the population pairs (*P* < 0.001). The linear relationship is depicted along with 95% confidence intervals.

The AMOVA showed that molecular variance was greatest among samples (74.6%) compared to populations (8.86%) and colonies (3.13%) (Table 3). Genetic differentiation (*F*_st_) under Nei for the data was 0.268 and found to be significantly different (*P* = 0.001) from the *F*_st_ value expected under HWE. *F*_st_ values between the populations ranged from 0.041 to 0.252 (supplementary table S2, Supplementary Material online). Steinkopf and No Heep were the most genetically similar populations (0.041). The isolation models suggest a marginal positive correlation between genetic distances and geographical distances (F = 31.77, beta = 0.7835, df = 2, 17, *P* = 0.048) and aridity index (F = 31.77, beta = 6.2386, df = 2, 17, *P* < 0.001). A second model testing isolation by environment using the residuals from a model of genetic distance against geographical distance identified a significant correlation (F = 12.16, beta = 0.37, df = 1, 18, *P* = 0.0026). Both models suggest a marginal correlation between genetic distance and geographical distance, but that isolation by the environment (IBE) explains the genetic differences between the populations (Fig. 2).

**Table 3:** Hierarchical analysis of molecular variance (AMOVA) for SNPs across five populations of *C. h. hottentotus*

Test	Variance (%)	Standard deviation	*P* value
Variations between samples	74.594	4.428	0.001*
Variations between colony	2.651	−0.111	0.514^NS^
Variations between population	7.506	1.048	0.151^NS^

Population and within-population colony assignments are predetermined based on provenance of collected samples. Significant results are in bold and marked with an asterisk (*). Nonsignificant results are denoted by NS.

### Aridity Gradient Shapes the Distribution of the Genetic Variation Among Populations

**Fig. 3.**
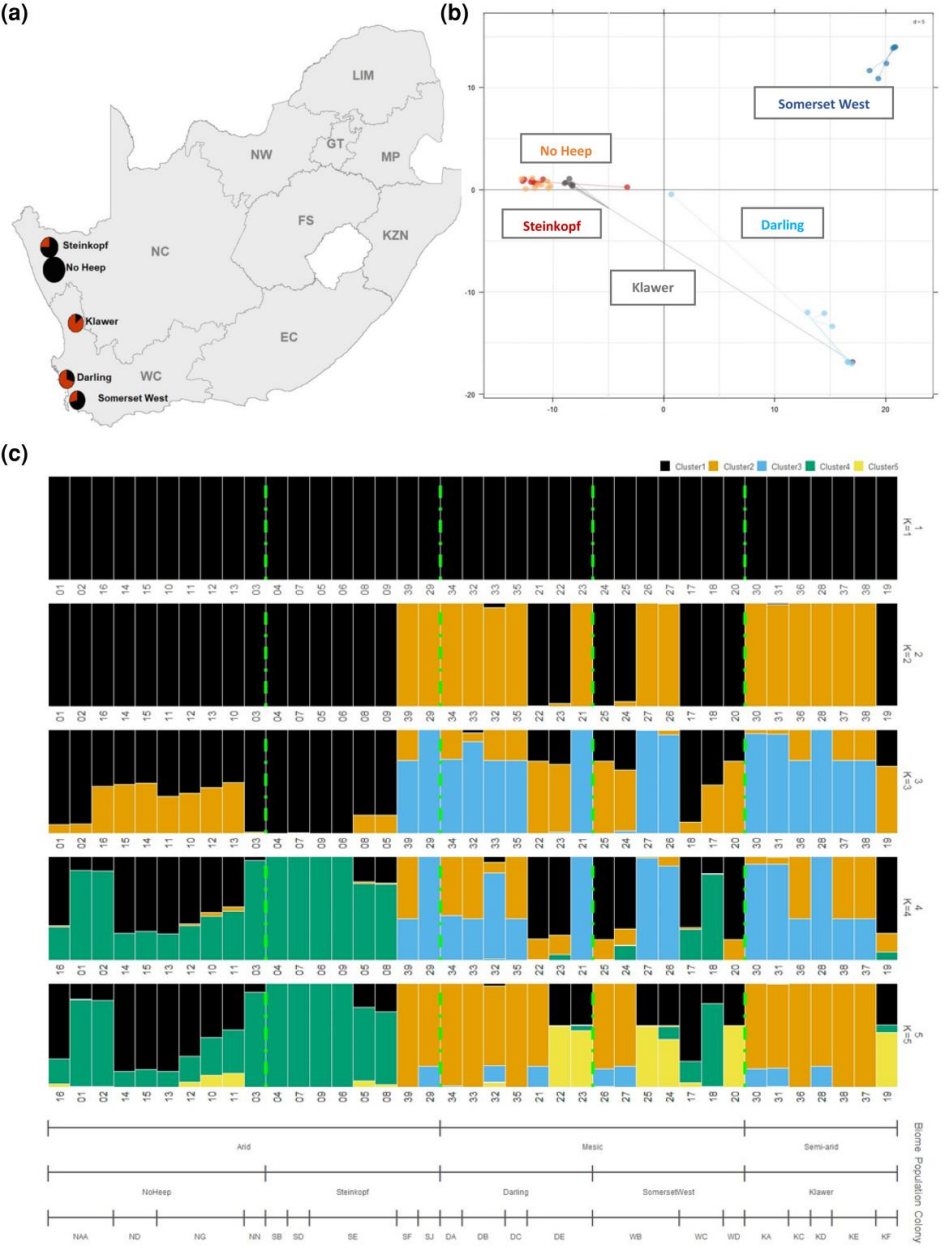
a) Geographical distribution of common mole-rat populations across the Northern Cape (NC) and Western Cape (WC) of South Africa sequenced in this study. Pie charts show the proportion of K = 1 and K = 2 present in each population according to STRUCTURE analysis. Number of individuals is represented by the size of pie charts. b) Principal component analysis of 3,540 SNP loci for 39 individuals of *C. h. hottentotus* across five populations, Darling, Somerset West, Klawer, No Heep, and Steinkopf. c) Bar plots showing the STRUCTURE results for the first five values of K. Cluster (K) colors are listed in the top right corner. Samples are grouped by biome (arid, mesic, and semi-arid), then by population (Steinkopf, No Heep, Klawer, Darling, and Somerset West) within that, and further separated by colony. Dashed lines separate populations. A box highlights the K value preferentially selected by STRUCTURE analyses (K = 2).

All clustering analyses (STRUCTURE, PCA, DAPC) concurred to show two main genetic clusters that encompassed (i) the two mesic populations, and (ii) the semi-arid and two arid populations. The STRUCTURE analysis showed two clusters (K = 2) with a mesic group encompassing the two mesic populations (Somerset West and Darling), and an arid group containing the two arid populations (Steinkopf and No Heep) and the intermediate, semi-arid population (Klawer). The assignment probability on an individual basis is listed in supplementary table S3, Supplementary Material online, and DeltaK values estimated using the Evanno methods (supplementary fig. S3, Supplementary Material online) from STRUCTURE results are shown in Fig. 3a and c.

The PCA results demonstrated the presence of two clusters, which is in line with the STRUCTURE results (Fig. 3b), and the DAPC results showed significant distinction between all five populations (supplementary fig. S4, Supplementary Material online). The results of the spatial PCA (sPCA) and Moran's I test supported the cluster analyses results and demonstrated the existence of a significant genetic structuring (*P* = 0.048) across the five populations. The sPCA did not detect a substructure within any of the groups.

## Discussion

Overall, the results from this study suggest that genetic diversity and population structure correlate with the environmental Aridity Index. The results demonstrate evidence of genetic differentiation between arid and mesic populations, represented in both the mitochondrial and nuclear data. Higher levels of genetic diversity are detectable in arid populations when compared to mesic populations based on the nuclear data.

### Evolutionary Relationships and Mitochondrial Variation Along an Aridity Gradient

A negative Tajima's *D* implies that a historic rapid population expansion (Dangalle et al. 2014) or a positive selection of certain alleles, known as selective sweep (De Jong et al. 2011), might have taken place. This is particularly evident in mesic-dwelling populations, suggesting that historically, *C. h. hottentotus* expanded from the north to the south and that mesic-dwelling individuals may have derived adaptations to mesic conditions, and arid populations retained ancestral haplotypes linked with arid adaption.

### Genetic Diversity and Structure Patterns Among Populations as a Function of an Aridity Gradient

HWE was not present in any of the populations apart from Klawer under the Chi-Squared test, thus suggesting the nuclear SNPs may be under selection, and that gene flow and genetic drift may be present, or that nonrandom mating is occurring, due to the presence of colonies within the populations (Abramovs et al. 2020). Indeed, the lack of HWE is further supported by the higher-than-expected presence of linkage disequilibrium found in all populations (Slatkin 2008). Due to the different ecological requirements in arid and non-arid environments, allele frequencies may be under different selection pressures, resulting in different genotypes (Orr 2005; Hoffmann and Willi 2008). However, due to the relatively small population sample sizes used in this study, there may be an increased effect of genetic drift. The smaller sample size of representative genomes may result in a random skew in allele frequencies, thus presenting with lower genetic variation than is present in the natural systems (Nei and Tajima 1981) and the fixation of certain alleles with no beneficial qualities in a population's adaptive response to its surrounding environment.

Heterozygosity was found to increase with aridity, i.e. as conditions become more arid, higher levels of heterozygosity are observed compared to mesic populations. We can thus infer that populations are variable and that habitat qualities such as aridity do play a role. Higher heterozygosity levels infer the presence of greater degrees of admixture in the arid region (Boca, et al. 2020). Greater admixture indicates more movement and greater occurrences of disperser events in arid regions between populations and colonies and thus, more gene flow. This pattern, however, is not consistent with the known examples of philopatry and dispersal differences between arid and mesic areas. The AFDH (Jarvis et al. 1994; Faulkes et al. 1997a; Spinks et al. 2000a; Spinks et al. 2000b; Jacobs et al. 2022) suggests that arid-dwelling *C. h. hottentotus* are more constrained in dispersal due to inhabiting a lower food-density environment. Therefore, movement has a greater energetic cost, thus, arid populations have significantly lower immigration and emigration rates and stronger social cohesion (Spinks et al. 2000a; Spinks et al. 2000b), in addition to being more xenophobic (Spinks 1998). Combining these factors is more likely to curtail the gene flow between populations, rather than promote it. Conversely, the AFDH states that ecological constraints in arid habitats promote the evolution of cooperative foraging and larger social groups. Indeed, these same arid conditions have been shown to drive arid-dwelling populations to dig longer burrow systems to compensate for low geophyte density (Spinks et al. 2000b) and arid populations are predicted to have increased numbers of individuals per colony, though this is yet to be demonstrated significantly (Spinks and Plagányi 1999; Spinks et al. 2000a). The greater colony size may increase genetic diversity, and larger burrow systems may potentially increase the number of encounters between the colonies and populations, providing opportunities for gene flow. Additionally, whilst there may be a case of reduced dispersal in arid populations, following rainfall events, individuals may disperse further than in the mesic populations, creating pathways for admixture (Lovegrove 1991; Spinks and Plagányi 1999). The connectivity of colonies in arid regions may be higher than expected, and there may be an effect on the distribution of colonies and individuals clustering around food sources of high geophyte abundance, thus leading to a greater chance of interactions.

The observed heterozygosity (*H*_o_) was higher than the expected (*H*_e_) for Steinkopf, No Heep, and Somerset West. However, when compared to the coefficient, uH_e_, which takes into account the small effective population sizes, the *H*_o_ was higher for Steinkopf and Somerset West only. Higher than expected levels of heterozygosity again implies that these populations are breaking the assumptions of HWE, and can indicate an isolate-breaking effect, which is the mixing of two previously isolated populations (Namipashaki et al. 2015). As Steinkopf and Somerset West are populations found at the most distal ends of the environmental continuum, they are likely experiencing the extremes of this environmental cline and, therefore, may be under greater levels of selection than the other populations (Spinks et al. 2000b). These populations also may represent range edge populations, and thus have the potential for greater movement into new areas, and higher levels of admixture with populations of neighboring subspecies compared to the other populations (No Heep, Klawer, and Darling) which demonstrate lower-than-expected levels of heterozygosity. Individuals from No Heep presented with little variation in heterozygosity compared to all other populations, potentially explained by genetic isolation due to the surrounding mountain ranges around No Heep. Darling and Somerset West populations (mesic dwelling) are located in a currently densely populated area with Cape Town in between, which might act as an anthropogenic barrier preventing admixture and gene flow. The arid regions of the Northern Cape are much more sparsely populated and thus have fewer barriers to dispersal and movement. The precise distributional ranges of the populations and more broadly the *Cryptomys* subspecies are not fully understood, and the presence of hybrid zones is unknown, but likely (Finn 2022).

The number of private alleles increased with both extreme high and extreme low AI. These results suggest that private alleles are accumulating in extreme arid and mesic environments, but less so in intermediate, semi-arid conditions. High numbers of unique loci in environmental extremes indicates selection for environmental adaptation, and thus these loci may be associated with thermal tolerance or osmoregulatory gene regions. A higher number of private alleles can also indicate past or recent genetic isolation in No Heep and Somerset West. *A*_r_ decreased significantly with an increase in AI, and thus was significantly lower in the mesic populations. Low *A*_r_ can indicate the presence of past genetic bottlenecks or reductions in population size and can suggest that these populations are particularly at risk of rapidly changing climates. Additionally, mole-rats have long generation lengths, so the individuals have a slower turnover and thus the movement of alleles within a population is much slower than other similarly sized rodents (Can et al. 2022). Different levels of linkage disequilibrium observed between the populations indicate the potential for populations to be independent, however the values did not differ greatly, so this remains uncertain. Linkage disequilibrium can also infer the presence of past bottleneck events occurring in the populations (Slatkin 2008) and may influence the heterozygosity results. Both of these findings indicate that the results from the program STRUCTURE need to be carefully considered (Pritchard et al. 2000), however the STRUCTURE analysis corroborated with PCA and DAPC and as a consequence we can infer that the STRUCTURE results can be considered in our determination of clusters.

### Aridity Gradient Shapes the Distribution of the Genetic Variation Among Populations

The isolation by environment model was significant implying that genetic distinction is potentially linked to environmental factors, namely Aridity Index (AI). The residual variation in genetic distance against geographic distance was explained by aridity index, thus isolating the impact of AI once the impact of distance and autocorrelation had been accounted for, further supporting the isolation by environment model. Genetic distance is virtually identical at Steinkopf and No Heep but differs more between Somerset West and Darling. These differences in genetic distance are reflected in the differences in the AI at these sites, thus supporting the idea that aridity may influence genetic distinction. However, as isolation by geographic distance was on the threshold of significance (*P* = 0.048), it is worth considering that geographical distance may be impacting genetic distance. Furthermore, the small sample sizes in this study may be reducing the impact of both factors. As the sample sites were located in a linear transect, geographic distance contributes to differences in aridity, so it remains difficult to tease apart the geographic and environmental impacts.

The results from the AMOVA indicate that genetic variance was greatest between individuals and lowest between colonies. The dispersal strategies of mole-rats naturally lead to high levels of relatedness between colonies. Dispersers move from a colony and migrate, likely to the next available colony, suggesting that colony proximity plays a role in genetic diversity (Greeff and Bennett 2000). This importance of colony proximity can lead to high levels of genetic homogeneity between colonies, with neighboring colonies containing relatives of each other (Spinks et al. 1999). Genetic variation between the populations was lowest indicating that population structures are not highly distinct and potentially not entirely independent. The high variation between individuals, however, suggests that relatively high levels of diversity are present in the species as a whole, with the presence of varied alleles that may or may not be linked to habitat adaptation. The high number of individuals from each population with genetically similar origins may again be due to the nontraditional, nonrandom mating system of the social common mole-rats.

The population statistic results are further supported by the cluster analyses and mitochondrial data, all of which suggest the presence of distinct mesic and arid clusters. The intermediate population, Klawer, share alleles with both arid and mesic clusters, but shares greater genetic similarities with the arid populations, which corresponds with the support for isolation by environment, as the AI for the site Klawer, is closer to the AI for the two arid populations, than the two mesic. These analyses demonstrate the multiple influences, proximity and environmental similarities, that effect gene flow and connectivity. Cluster analyses can be deceptive with populations that are not truly independent, for example, those found on a continuum or along an environmental cline (Lombaert et al. 2018). Thus, multiple cluster analyses were used in order to strengthen the results. Additionally, the known provenance of the individuals used in this study, and the colonial nature of this species, can also provide support for the results from these analyses.

### Limitations and Future Work

The populations used in this study form a linear continuum and therefore the genetic diversity exhibited in our results reflects diversity along one dimension. As such, future work would benefit from the sampling of populations that vary in longitude as well as latitude. Further work on population structure and dispersal, investigating populations closer in proximity, is needed. A follow-up study using increased numbers per population from many colonies would provide a better representation of the natural populations that exist in the wild. This present study provides a baseline against which to unravel different colony roles, such as queens and breeding males to provide an understanding of the individual colony structures in each population, which may infer and explain the movements, or lack thereof between colonies in each population as well as further understand how they differ in the different habitat types. Finally, the next steps from this study would be to investigate variation in target loci for environmental and thermal adaptation to determine specific environmental-based differences between the populations.

Nonrandom mating can raise the observed frequency of homozygous alleles in the overall population to higher than expected under HWE, known as the Wahlund effect (Sinnock 1975). The mating system of social mole-rats creates an effect of subpopulations, or colonies, in which only certain individuals, the queen and breeding males, will mate with each other and produce offspring, forming colonies of closely related sibling and parent family groups (Bennett and Faulkes 2000). It is thus possible that incest avoidance is operational in common mole-rats and the evidence of inbreeding may simply result from sampling family members. The population inbreeding and heterozygosity values did not differ greatly when only one individual per colony was used. Nevertheless, the use of *F*_is_ can be misleading when considering colonial species that have nontraditional mating systems, as present in common mole-rats, confounding the inbreeding coefficient. Often, in the case of African mole-rats, the dispersers are (potential) breeding males, so these are likely to be the more genetically distinct individuals in a colony and increase genetic diversity (Torrents-Ticó et al. 2018). “Floater males” are also common in mole-rat populations, and thus paternities in colonies may not be attributed to any colony residents (Bishop et al. 2004). As breeding individuals were not included extensively in this study and may be important in further studies to gain a more in-depth picture of genetic diversity of common mole-rats. Interestingly, the Klawer population had two colonies, F and G, with two queens each. The presence of multiple reproductive females in one colony is rare for *C. h. hottentotus*. Multiple queens per colony have been previously encountered in mesic sites, but has, as of yet, never been formally reported in arid sites (Spinks et al. 2000a). Klawer is classified semi-arid and in colony G both queens had the same haplotype, which is insufficient to establish the matrilineal relatedness. In colony F, the haplotypes were different, suggesting that the two queens come from different maternal lineages, highlighting the importance of exploring breeding and nonbreeding individuals.

### Conclusions

Overall, our results demonstrate a relationship between environment and genetic diversity and its distribution across populations at a regional scale. This study shows that mole-rats in arid environments have increased genetic diversity and greater connectivity between populations than mesic populations. Genetic diversity may be greater in arid populations as a consequence of resource distribution and dispersal patterns, as well as human settlement distribution. Mesic populations are therefore potentially more at risk in a changing climate due to their low genetic diversity and low initial allelic composition. The potential for loss of distributional range for this species could thus be high, and therefore further investigation of their population genetics, and specific adaptations may influence conservation policies that need to be put in place to ensure the survival of this species and the various potential morphotypes that exist.

## Methods

### Study Species

Bathyergid mole-rats vary in social strategies from solitary to truly social or eusocial, living in colonies of closely related family groups (Bennett et al. 1990). Populations of the cooperatively breeding common mole-rats (*C. h. hottentotus*) present an ideal model system for the study of mammalian population genetics as the distributional range of this subspecies spans from the arid succulent Karoo in the Northern Cape through to the mesic Fynbos of the Western Cape (Spinks et al. 2000a).

### Study Site

The sites were specifically selected to represent an aridity gradient based on an Aridity Index (AI), a numerical indicator of the degree of dryness of the climate at a given location (UNEP, 1992). The AI for the study populations was calculated from climatic data (ranging from the years 1981 to 2020) retrieved from the ERA5-Land of the European Centre for Medium-Range Weather Forecasts, created by the Copernicus Climate Change Service (Muñoz-Sabater et al. 2021) with a spatial resolution of 0.1° by 0.1°. Monthly averaged temperature (*T*_air_ in °C), total precipitation (*t*_p_ in m), and dew point temperature (d2m in °C) were used. These combined data were used to calculate the annual aridity index (AI) (equation (1)). Where *t*_p_ is directly obtained from ERA5-Land and potential evapotranspiration (PET) is calculated from the Romanenko estimation (equation (2)) (Romanenko 1961). For equation (2), relative humidity (RH) was calculated from ERA5-Land d2m (equation (3)).


(1) $$\mathrm{AI}\;\;=\;\;\frac{t_{p}}{\mathrm{PET}}\;$$

(2) $$\mathrm{PET}\;=\;0.00006\times (100\;\text{–}\;\mathrm{RH})\times (25+T_{\mathrm{air}}{)}^{2}\;$$

(3) $$\mathrm{RH}=100\;\times \;10^{7.591386\;\left(\frac{d2m}{d2m\;\;+\;\;240.7263}\;-\frac{T_{\mathrm{air}}}{T_{\mathrm{air}}\;\;+\;\;240.7263}\right)}$$

Aridity classifications and corresponding AI values, as outlined by UNESCO (1979) and UNEP (1992), state that where PET is greater than *t*_p_, the climate is considered to be arid (Colantoni et al. 2015). AI values at each of the five sites used in this study are listed in Table 4 and have been selected to range in AI across different aridity classifications. The two municipalities of Steinkopf and No Heep are considered arid populations, Klawer a semi-arid/intermediate, and the municipalities of Darling and Somerset West mesic populations.

**Table 4:** List of five sites, sample size for both the mitochondrial dataset (cyt-b), and whole genome dataset (WG) with collection coordinates for individuals of common mole-rats, recent Aridity Index values (from the year 2020) for each site based on climate data taken from ERA5-Land dataset to two decimal places, and aridity classifications are listed

Site	Sample size: cyt-b/WG	Latitude	Longitude	Aridity index	Aridity classification
Steinkopf	7/8	−29.34531	17.7872	0.04	Arid
No Heep	9 (+1 Kamieskroon)/10	−30.04253	17.95852	0.07	Arid
Klawer	11/7	−31.7013476	18.7446117	0.11	Semi-arid
Darling	6/7	−33.406573	18.417538	0.42	Mesic
Somerset West	4/7	−34.035613	18.799499	0.86	Mesic

### Sample Collection

Samples of common mole-rats were collected from 39 individuals across five sites, from between seven and four colonies per site (supplementary table S1, Supplementary Material online). Mole-rat individuals were trapped using Hickman live traps (Hickman 1979) inserted into tunnels located under mounds and were baited with sweet potato. All sites have been previously documented as having common mole-rats (Spinks et al. 2000a; Visser et al. 2019; Hart et al. 2023). The animals used in this study were maintained in captivity for between 22 and 28 days prior to being euthanized using an overdose of isoflurane in line with strict veterinary procedures. Individuals were collected from multiple colonies within each of the five populations (two arid, one semi-arid, and two non-arid). The Animal Use and Care Committee of the University of Pretoria evaluated and approved experimental protocol (ethics clearance no. NAS016/2021) and DAFF section 20 approval (SDAH-Epi-21031811071). Tissues were then harvested by cutting open the abdomen and chest cavity biceps femoris muscle tissue was extracted and fixed in 70% ethanol and stored at −20 °C prior to extraction.

### DNA Extraction

Extractions were first carried out using the QIAGEN Dneasy Blood and Tissue Kit as specified by the manufacturer. The QIAGEN Kit extraction method yielded low DNA concentration values, thus a second set of extractions were carried out on the samples using a standard salt/chloroform extraction protocol as outlined by Müllenbach et al. (1989). A full list of analyses performed, and sample information is given in supplementary table S4, Supplementary Material online.

### Mitochondrial DNA Sequencing

PCR amplification of the entire cytochrome b gene (1,140 bp) was carried out using primers (forward: L14724, reverse: H15915) described previously in Faulkes et al. (1997a, 1997b). Sanger sequencing was carried out in both directions using the Eurofins Genomics Value Read provided by Eurofins MWG Operon Inc. (Eurofins Scientific, Luxembourg, Luxembourg) to obtain complimentary and partially overlapping strands for 39 individuals.

Sequence alignment was performed manually in Mesquite version 3.70 (Maddison and Maddison 2021). Sequences of other African mole-rat species were obtained from GenBank (Clark et al. 2016), and used as references for sequence alignment, namely *Heterocephalus glaber* accession no. MT8453841 (Zemlemerova et al. 2021), *Fukomys damarensis* accession no. AF012223.1 (Faulkes et al. 1997a), and *C. h. hottentotus* accession no. MH186559.1 (Visser et al. 2019). One additional *C. h. hottentotus* sequence was added to the database from Kamieskroon (30°13′09.0″S 17°56′20.1″E), a site located near No Heep. This sequence was obtained from an old sample collected in 2016 for a previous study held at Queen Mary University of London.

### Analysis of Evolutionary Relationships and Mitochondrial Variation Along an Aridity Gradient

The mitochondrial data consisted of sequences from 39 samples (supplementary table S4, Supplementary Material online and one additional sample from Kamieskroon), and one reference sequence for *F. damarensis* as an outgroup. Phylogenetic trees were constructed using Molecular Evolutionary Genetic Analysis version 11 (MEGA11; Tamura et al. 2021; Stecher et al. 2020). The Maximum Parsimony (MP) method for analysis of taxa (Nei and Kumar 2000) with the Subtree-Pruning-Regrafting (SPR) algorithm (Nei and Kumar 2000) and the Maximum Likelihood (ML) method using Hasegawa–Kishino–Yano model (Hasegawa et al. 1985) were used. To estimate the robustness of the tree topology, bootstrap analysis was conducted with 1,000 replicates of the dataset (Felsenstein 1985).

A Minimum Spanning Network (Bandelt et al. 1999) analysis was used to determine the haplotypes present in the mtDNA full sequence dataset containing 39 sequences. PopART version 1.7 (Leigh and Bryant 2015) was used to tag sequences by population, which were then grouped into three biomes according to respective Aridity Index (Steinkopf, No Heep, Kamieskroon as arid, Klawer as semi-arid, Darling and Somerset West as mesic). A hierarchical (nested) AMOVA (Excoffier et al. 1992) was performed to calculate Ф-statistics and identify geographical patterns of genetic differentiation between haplotypes. Significance was calculated for 1,000 permutations. Values for nucleotide diversity (π), defined as average pairwise differences per site for all sequences, and Tajima's *D* statistic for population neutrality (Tajima 1989) were obtained, and a map of the geographical distribution of haplotypes was drawn from haplotypes data in PopART.

### UCE Targeted Sequence Capture

Probes were designed and synthesized by Daicel Arbor Biosciences, Ann Arbor, MI, USA. Genome libraries were prepared using the NEBNext Ultra II FS DNA Library Prep Kit for Illumina TruSeq (New England BioLabs, Inc., Ipswich, MA, USA), as per the manufacturer's recommended protocol for fragmentation system DNA library preparations with inputs ≥ 100 ng. Fragment sizes of between 300 and 500 bp were required and therefore, Size Selection protocol using AMPure XP Beads was included, and specific conditions within the protocol were selected based on this fragment size range as per the Library Prep Kit protocol, and as requested by Daicel Arbor Biosciences, Ann Arbor, MI, USA. Libraries were pooled in equimolar proportions into six pools prior to shipping to Daicel Arbor Biosciences. Total DNA was quantified via a spectrofluorimetric assay, and the mass of molecules per library was quantified via a quantitative PCR assay. Captures were performed following the myBaits v5.02 protocol (Daicel Arbor Biosciences, Ann Arbor, MI, USA) using myBaits UCE Tetrapods 5Kv1 baits with an overnight hybridization and washes at 65 °C. Post-capture, the reactions were amplified for ten cycles and were quantified again with a spectrofluorimetric assay. The captures were pooled in approximately equimolar ratios (supplementary table S5, Supplementary Material online). Samples were sequenced on the Illumina NovaSeq 6000 platform on a partial S4 PE150 lane to approximately 1 Gbp per library. Demultiplexed fastq data were returned with successful sequence reads for 39 samples (supplementary table S4, Supplementary Material online).

### Bioinformatics for UCE SNP Loci

Using the Python package, PHYLUCE Version 1.7.2 (Faircloth 2016), through the Linux distribution, Ubuntu version 22.04.1 (Sobell 2015), UCE data were processed, contigs assembled and loci aligned as per the protocol outlined by Faircloth (2016). Sequences were trimmed of adaptor contamination and low-quality bases using Illumiprocessor. Cleaned reads were inspected with fastQC to ensure only high-quality reads remained. Contigs from each individual were subsequently assembled from the cleaned reads to a reference set of UCE loci using the bwa-meme algorithm for each sample against the composite reference. Alignment included cleaning, validating, and marking duplicates that were then removed in the resulting Binary Alignment/Map (BAM) file. The reference genome used was from *F. damarensis* since no genomes have been sequenced within the genus *Cryptomys*. Extracted contigs were taken for each UCE locus, and depth and quality were assessed. The mapped reads were indexed. Using the probes to create a bed file, through bedtools to restrict to target regions only. Depth values were extracted, and sequences filtered to only select SNPs for each of the UCE loci. Sequences were filtered using VCFtools, creating a variant call format (VCF) file. The VCF file was filtered to retain a minimum genotype depth of 5 and a maximum genotype depth of 15 (mean depth for 50% and 80% of individuals) for SNPs successfully genotyped in ≥80% of the individuals. The parameters created a complete matrix with a minimum genotype quality (GQ) of 20. The SNPs were then pruned to only one SNP per locus to prevent extreme effects of linkage disequilibrium and to provide a set of independent markers to compete the VCF file.

### Genetic Diversity and Structure Analyses Among Populations Along an Aridity Gradient

Calculations and statistical analyses were performed using the statistical software R version 4.2.2 (R Core Team 2021). We compared population diversity among population using eight estimates of genetic diversity and genetic structure (Table 2) available in *adegenet* (Jombart 2008) and *hierfstat* (Goudet 2005) R packages. We test for deviations from the HWE for each population by both a Chi-Squared test and Monte Carlo permutation test with 1,000 replicates. Linkage disequilibrium values (*r*^2^_D_) were also determined for each population using 1,000 replicates, and for each pair of loci, to create an average value for our UCE loci. We used estimates of expected and observed heterozygosity (*H*_e_ and *H*_o_, respectively) and unbiased expected heterozygosity (uH_e_) to metrics of population-level genetic diversity, uH_e_ is defined by equation (4). We also estimated rarefied allele richness (*A*_r_), and the number of private alleles unique to each site as proxies of genetic diversity. The inbreeding coefficient (*F*_is_), calculated using equation (5), measured the proportion of the variance for each individual within a subpopulation and it is typically used as a proxy of gene flow among subpopulations, with high values of *F*_is_ implying low gene flow levels. Linear mixed effects models with binomial distributions (heterozygosity and *F*_is_) and general linear models (allelic richness, private allele number) were used to determine differences between the populations for each of the parameters and this was carried out on the full dataset and a subset of one individual per colony present in the study to explore the effects of family groups.


(4) $$uH_{e}=\;H_{e}\times \frac{2\times \mathrm{Population}\;\mathrm{size}}{2\times \mathrm{Population}\;\mathrm{size}-1}$$

(5) $$F_{\mathrm{is}}=\frac{1-\mathrm{mean}(H_{o})}{\mathrm{mean}(uH_{e})}$$

### Analysis of Genetic Structure in Response to Aridity

An AMOVA was carried out based on 999 replicates to assess the variation in genetic diversity both among the populations and within populations, through variation between individuals (samples), and variation between the different colonies found within a population. Genetic differentiation across the populations (*F*_st_) was assessed using the R package *hierfstat* (Goudet 2005). *F*_st_ was calculated under a Nei distribution (Nei 1978). We tested for isolation by distance (IBD) and IBE (Wang et al. 2013), in our study system through models using the pairwise Nei's genetic distances, geographic distances based on longitude and latitude for each site, and a distance matrix of Aridity Index for each site (environmental distance) (Table 4). Using the R package *spaMM*, isolation patterns were first tested using a linear model using matrices for genetic distance as the dependent variable, and geographic distance and environmental distance as dependent variables. A second test was carried out by extracting residuals from a model of genetic distance against geographical distance, and modeling this against environmental distance.

### Cluster Analysis of Populations Along an Aridity Gradient

We test for clustering patterns and whether aridity underlies observed patterns by applying an equilibrium-based model (STRUCTURE) and nonequilibrium-based clustering models (PCA and DAPC). The VCF file was converted into STRUCTURE file format using PGDSpider version 2.1.1.5 (Lischer and Excoffier 2012). STRUCTURE version 2.3.4 software (Pritchard et al. 2000) was first used to carry out clustering analyses. STRUCTURE uses Bayesian model-based methods that presume HWE and linkage disequilibrium, analyzing the individual-level multilocus genotypes and assigning individuals to clusters based on their assignment probability. We set a burn-in period of 50,000 iterations and 200,000 iterations with Markov Chain Monte Carlo (MCMC). An admixture model was assumed, and geographic location was considered in the analysis. K values ranged from 1 to 21, and ten runs per K value. We applied the Evanno method (Evanno et al. 2005) to select the most likely K value and as implemented and visualized using the package *pophelper* in R (Francis 2017).

STRUCTURE analyses can be confounded by populations that are not truly independent, for example, with populations found along an environmental cline. To strengthen the results, further clustering analyses were carried out using the *adegenet* R package, to combine different methodological approaches and compare results from equilibrium-based models (STRUCTURE) and nonequilibrium-based models (PCA and DAPC). A principal component analysis (PCA) was used to confirm the clustering of individuals into distinctive groups. A PCA synthesizes the multilocus data to create a multivariate space where individuals are mapped according to their similarities and determine principal components that best explain the data. Finally, a distance-based discriminant analysis of principal components (DAPC) was used. DAPC build genetic clusters from genetic data by combining the alleles. Neither the PCA or the DAPC require assumptions of HWE or linkage disequilibrium. The most likely K value for the DAPC was selected using the Bayesian information criterion (BIC) and the optimal K value identified through an accompanying decrease in the BIC.

Due to the evident spatial patterns in the PCA, a spatial PCA (sPCA) was carried out using *adegenet*. A sPCA uses Moran's I to incorporate spatial autocorrelation between samples so that genetic structure can be modeled without a priori population assignment and allows for visualizing population structure in space. This analysis method, similar to a PCA, does not assume HWE or linkage disequilibrium. Longitude and latitude for each colony within a population were used, and unique individual coordinates were created with the “jitter” function in R to develop unique *x* and *y* values. sPCA eigenvalues were tested for global and local structures, which correspond to positive spatial autocorrelation between individuals, such as patches, clines and intermediates, and strong genetic differences between neighbors, using 1,000 Monte Carlo tests replicates.

## Supplementary Material

evae144_Supplementary_Data

## Data Availability

All data are available as an electronical supplementary file.
