# Supplementary material for: Patterns of Genetic Diversity and Gene Flow Associated With an Aridity Gradient in Populations of Common Mole-rats, Cryptomys hottentotus hottentotus
Source: Genome Biol Evol. 2024 Jul 2;16(7):evae144. doi: 10.1093/gbe/evae144 (PMC11258414; doi:10.1093/gbe/evae144)
Supplement: evae144_Supplementary_Data [file evae144_supplementary_data.zip › Genetics Supplementary_revised_final_2.docx]

**Supplementary Information for:**

**Patterns of genetic diversity and gene flow associated with an aridity gradient in populations of common mole-rats, *Cryptomys hottentotus hottentotus***

**Hana N. Merchant^1,2^*, Anastasia Ivanova^2^, Daniel W. Hart^3^, Cristina García^1^, Nigel C. Bennett^4^, Steven J. Portugal^1,5^ and Chris G. Faulkes^2^**

**^1^Department of Biological Sciences, School of Life and Environmental Sciences, Royal Holloway University of London, Egham, Surrey TW20 0EX, United Kingdom**

**^2^School of Biological and Behavioural Sciences, Queen Mary University of London, London, United Kingdom**

**^3^Department of Zoology and Entomology, University of Pretoria, Pretoria, Gauteng, South Africa**

**^4^Mammal Research Institute, Department of Zoology and Entomology, University of Pretoria, Pretoria, Gauteng, South Africa**

**^5^Department of Biology, University of Oxford, Oxford OX1 3SZ, United Kingdom**

***Correspondence to:** [**Hana.Merchant.2020@live.rhul.ac.uk**](mailto:Hana.Merchant.2020@live.rhul.ac.uk)

**Figure S1.** Phylogenetic trees based on mitochondrial cytochrome b data. **a)** Maximum parsimony phylogenetic tree of the observed haplotypes and **b)** maximum likelihood phylogenetic tree of the observed haplotypes. Node numbers indicate bootstrap values for 1,000 replicates. Populations are colour coded and *Fukomys damarensis* is used as an outgroup. See Table S1 for haplotype naming system. A Maximum Parsimony tree (a) showed differentiation between arid and semi-arid populations (Steinkopf, No Heep, Kamieskroon, Klawer), and mesic populations (Darling, Somerset West). The consistency index (CI), retention index (RI), and rescaled consistency index (RC) were high for all sites (CI = 0.89, RI = 0.86, RC = 0.76), as well as for parsimony-informative sites (CI = 0.68, RI = 0.86, RC = 0.58), although for parsimony-informative sites the values are lower. The bootstrap values were relatively high (>80) for all the nodes that denote divergence between populations, except for the node between Steinkopf and No Heep, where the bootstrap values were low (<50). Within populations, divergence between haplotypes was lower, yet still ∼70 for arid and semi-arid populations, and higher confidence (>84) for mesic populations. Similarly, the Maximum Likelihood tree using a Hasegawa-Kishino-Yano model of evolution (b) showed differentiation between arid and semi-arid populations, and mesic populations, however, the statistical support for most of the nodes in the resulting phylogenetic tree was low, as few nodes had values of >70. These include differentiation between Darling and Somerset West, as well as between haplotypes within these populations. Differentiation between No Heep and Klawer was found to be robust, as well as the differentiation of two lineages within No Heep. All other nodes, however, showed very low bootstrap values and are not well supported.

**Table S1.** Twenty haplotypes identified from mitochondrial cytochrome b data across six populations of *C. h. hottentotus*. The table shows haplotype number, haplotype names, the population in which they are found as well as the number of samples that belong to this haplotype. Haplotype name reads as follows: “Number Population Colony1 Colony2 Colony3”, etc. “.Q” or “.BM” after colony name represents queen or breeding male respectively (e.g. 13 Klawer C F.Q C.Q reads “Number 13, population Klawer, colony C non-breeder, colony F queen, colony C queen).

| **Haplotype number** | **Haplotype name** |  | **Population** | **Number of samples** |
| --- | --- | --- | --- | --- |
| 1 | Steinkopf D B.Q C No Heep B.BM |  | Steinkopf, No Heep | 4 |
| 2 | Steinkopf J F F E.Q |  | Steinkopf | 4 |
| 3 | No Heep F |  | No Heep | 1 |
| 4 | No Heep W B.BM |  | No Heep | 2 |
| 6 | No Heep N.Q |  | No Heep | 1 |
| 7 | No Heep A.Q |  | No Heep | 1 |
| 5 | No Heep Y N G |  | No Heep | 3 |
| 9 | No Heep D |  | No Heep | 1 |
| 11 | Klawer F |  | Klawer | 1 |
| 16 | Klawer A.Q |  | Klawer | 1 |
| 12 | Klawer D E E |  | Klawer | 3 |
| 15 | Klawer F.Q G.Q G.Q |  | Klawer | 3 |
| 13 | Klawer C F.Q C.Q |  | Klawer | 3 |
| 18 | Darling C |  | Darling | 1 |
| 17 | Darling D.Q |  | Darling | 1 |
| 19 | Darling A E.BM E.Q B.Q |  | Darling | 4 |
| 8 | Kamieskroon |  | Kamieskroon | 1 |
| 22 | Somerset West A C |  | Somerset West | 2 |
| 23 | Somerset West D |  | Somerset West | 1 |
| 21 | Somerset West B |  | Somerset West | 1 |

**Figure S2.** Heterozygosity estimates of each of the populations are superimposed onto a neighbour-joining tree from genome-wide genetic distance estimates (F_st_) (branch lengths in units of substitutions per bp). Clades are coloured according to biome type, arid (red), semi-arid (grey) and mesic (blue).

**Table S2.** Matrix of genetic differentiation (F_st_) values under Nei assumptions for each of the five populations of *C. h. hottentotus*. F_st_ values range from 0 to 1, with genetic distinction being lowest at 0, and greatest at 1.

|  | **Steinkopf** | **No Heep** | **Klawer** | **Darling** | **Somerset West** |
| --- | --- | --- | --- | --- | --- |
| **Steinkopf** |  |  |  |  |  |
| **No Heep** | 0.041 |  |  |  |  |
| **Klawer** | 0.093 | 0.076 |  |  |  |
| **Darling** | 0.22 | 0.211 | 0.137 |  |  |
| **Somerset West** | 0.252 | 0.244 | 0.197 | 0.148 |  |

**Table S3.** Cluster assignment probability for each individual based on STRUCTURE analyses when K = 2.

|  | **Cluster1** | **Cluster2** |
| --- | --- | --- |
| **H10** | 1.0000 | 0.0000 |
| **H11** | 1.0000 | 0.0000 |
| **H14** | 1.0000 | 0.0000 |
| **H15** | 1.0000 | 0.0000 |
| **H17** | 1.0000 | 0.0000 |
| **H18** | 1.0000 | 0.0000 |
| **H2** | 1.0000 | 0.0000 |
| **H20** | 1.0000 | 0.0000 |
| **H21** | 1.0000 | 0.0000 |
| **H23** | 0.9999 | 0.0001 |
| **H24** | 1.0000 | 0.0000 |
| **H26** | 1.0000 | 0.0000 |
| **H28** | 1.0000 | 0.0000 |
| **H29** | 1.0000 | 0.0000 |
| **H30** | 1.0000 | 0.0000 |
| **H33** | 1.0000 | 0.0000 |
| **H36** | 1.0000 | 0.0000 |
| **H37** | 1.0000 | 0.0000 |
| **H38** | 0.9947 | 0.0053 |
| **H40** | 0.9998 | 0.0002 |
| **H41** | 0.0000 | 1.0000 |
| **H42** | 0.9977 | 0.0023 |
| **H43** | 0.9693 | 0.0307 |
| **H44** | 0.9558 | 0.0442 |
| **H45** | 0.9988 | 0.0012 |
| **H46** | 0.0054 | 0.9946 |
| **H47** | 0.0000 | 1.0000 |
| **H48** | 0.0000 | 1.0000 |
| **H5** | 0.0000 | 1.0000 |
| **H53** | 0.0012 | 0.9988 |
| **H54** | 0.0096 | 0.9904 |
| **H55** | 0.0430 | 0.9570 |
| **H56** | 0.0000 | 1.0000 |
| **H61** | 0.0000 | 1.0000 |
| **H62** | 0.0000 | 1.0000 |
| **H64** | 0.0000 | 1.0000 |
| **H67** | 0.0000 | 1.0000 |
| **H68** | 0.0000 | 1.0000 |
| **H8** | 0.0000 | 1.0000 |

**Figure S3.** DeltaK values for the STRUCTURE analysis. This value is used to select the optimal number of groups in the STRUCTURE analysis (i.e. K). The best K value is that with higher Delta K, that in our case is two.

**Figure S4.** Distribution of individuals as explained by the first two canonical variates (CV1 and CV2) derived from 3,540 SNP loci for 39 individuals of *C. h. hottentotus* across five populations, Darling, Somerset West, Klawer, No Heep and Steinkopf.

**Table S4.** List of samples that were successfully sequenced. Samples used in genomic targeted capture (UCEs) and the mitochondrial cytochrome b gene (mtDNA) are indicated with a Y in the respective columns. Sample details are listed for population, colony, sex, age and breeding status where information was available. For myBaits sequences, indexes are listed.

**(SEE EXCEL FILE)**

**Table S5.** Details for each of the six pools (total of eight samples per pool) created for sequencing by Arbor Biosciences. Indexes were grouped to avoid overlap in primer sequences. Indexes marked with an asterisk (*) pertain to indexes from a NGS UDI Primer Set 96-1 (Eurofins Scientific, Luxembourg, Luxembourg), and all other indexes are from the NEBNEXT Oligos for Illumina (Primer Set 3) (New England BioLabs, Inc., Ipswich, MA, USA). TapeStation concentrations for all samples and Qubit concentrations for most samples are listed and used to calculate the mass and volume added to each pool to create equal concentrations per sample. Well position in the plate is listed.

| **Sample** | **Index** | **Tape conc** | **Qubit conc** | **Mass (ng)** | **Volume (µl)** | **Well position** |
| --- | --- | --- | --- | --- | --- | --- |
| **H5** | A12 | 13.7 | 37.4 | 383.6 | 12.56 | A1 |
| **H8** | B12 | 17.9 | 40.6 | 501.2 | 9.62 | A1 |
| **H10** | C12 | 6.15 | - | 172.2 | 28 | A1 |
| **H11** | D12 | 16.3 | 47.6 | 456.4 | 10.57 | A1 |
| **H64** | C6 | 6.77 | 13.5 | 189.56 | 25.45 | A1 |
| **H14** | F12 | 6.19 | 15.6 | 173.32 | 27.72 | A1 |
| **H67** | F6 | 13.9 | 21 | 389.2 | 12.39 | A1 |
| **H17** | H12 | 15.5 | - | 434 | 11.11 | A1 |
| **TOTAL MASS (µg)** | **1.3776** |  |  |  |  |  |
| **TOTAL VOLUME (µl)** | **137.42** |  |  |  |  |  |
| **H4** | H10* | 11.9 | 31.4 | 333.2 | 11.02 | B1 |
| **H53** | B7 | 5.84 | 8.58 | 163.52 | 22.22 | B1 |
| **H21** | C11 | 15.8 | 32.8 | 442.4 | 8.24 | B1 |
| **H23** | D11 | 6.01 | 29.8 | 168.28 | 21.71 | B1 |
| **H24** | E11 | 9.3 | 38.2 | 260.4 | 14 | B1 |
| **H3** | G10* | 4.65 | - | 130.2 | 28 | B1 |
| **H26** | G11 | 15.1 | 32.4 | 422.8 | 8.62 | B1 |
| **H28** | H11 | 8.17 | 34.4 | 228.76 | 15.91 | B1 |
| **TOTAL MASS (µg)** | **1.0416** |  |  |  |  |  |
| **TOTAL VOLUME (µl)** | **129.72** |  |  |  |  |  |
| **H30** | A10 | 6.03 | 49.2 | 168.84 | 28 | C1 |
| **H55** | C7 | 8.96 | 27 | 250.88 | 18.79 | C1 |
| **H37** | C10 | 7.41 | 27.6 | 207.48 | 22.76 | C1 |
| **H40** | D10 | 6.67 | 12.6 | 186.76 | 25 | C1 |
| **H42** | E10 | 9.59 | 27.6 | 268.52 | 17.61 | C1 |
| **H43** | F10 | 6.59 | 18.8 | 184.52 | 25.69 | C1 |
| **H44** | G10 | 7.6 | 33.8 | 212.8 | 22.22 | C1 |
| **H46** | H10 | 13 | 24.2 | 364 | 12.96 | C1 |
| **TOTAL MASS (µg)** | **1.35072** |  |  |  |  |  |
| **TOTAL VOLUME (µl)** | **173.03** |  |  |  |  |  |
| **H48** | A9 | 11.4 | 37.2 | 319.2 | 20.74 | D1 |
| **H54** | B9 | 14 | 29.6 | 392 | 16.87 | D1 |
| **H29** | C9 | 8.43 | 39.6 | 236.04 | 28 | D1 |
| **H36** | D9 | 21 | 30.4 | 588 | 11.24 | D1 |
| **H38** | E9 | 10.2 | 28.2 | 285.6 | 23.14 | D1 |
| **H41** | F9 | 20.2 | 34.4 | 565.6 | 11.69 | D1 |
| **H45** | G9 | 10.3 | 20.2 | 288.4 | 22.95 | D1 |
| **H47** | H9 | 12.1 | 54.6 | 338.8 | 19.51 | D1 |
| **TOTAL MASS (µg)** | **1.88832** |  |  |  |  |  |
| **TOTAL VOLUME (µl)** | **154.14** |  |  |  |  |  |
| **H6** | A10* | 9.24 | 38 | 258.72 | 20.44 | E1 |
| **H2** | B8 | 23.2 | 41.2 | 649.6 | 8.16 | E1 |
| **H18** | C8 | 7.2 | 23.6 | 201.6 | 26.17 | E1 |
| **H7** | B10* | 6.76 | 14 | 189.28 | 28 | E1 |
| **H20** | E8 | 11.5 | 26.8 | 322 | 16.47 | E1 |
| **H33** | F8 | 15.7 | 35.8 | 439.6 | 12.07 | E1 |
| **H68** | G6 | 8.78 | 29.6 | 245.84 | 21.5 | E1 |
| **H15** | H8 | 10.6 | 26.2 | 296.8 | 17.83 | E1 |
| **TOTAL MASS (µg)** | **1.51424** |  |  |  |  |  |
| **TOTAL VOLUME (µl)** | **150.64** |  |  |  |  |  |
| **H56** | D7 | 0/0.206 | 2.58 | 72.24 | 28 | F1 |
| **H61** | A6 | 0.226/0.584 | 3.68 | 103.04 | 28 | F1 |
| **H62** | A8 | 0.925/1.45 | 20.6 | 176.4 | 28 | F1 |
| **TOTAL MASS (µg)** | **0.35168** |  |  |  |  |  |
| **TOTAL VOLUME (µl)** | **84** |  |  |  |  |  |
