# Supplementary figures and images for: Patterns of Genetic Diversity and Gene Flow Associated With an Aridity Gradient in Populations of Common Mole-rats, Cryptomys hottentotus hottentotus
Source: Genome Biol Evol. 2024 Jul 2;16(7):evae144. doi: 10.1093/gbe/evae144 (PMC11258414; doi:10.1093/gbe/evae144)

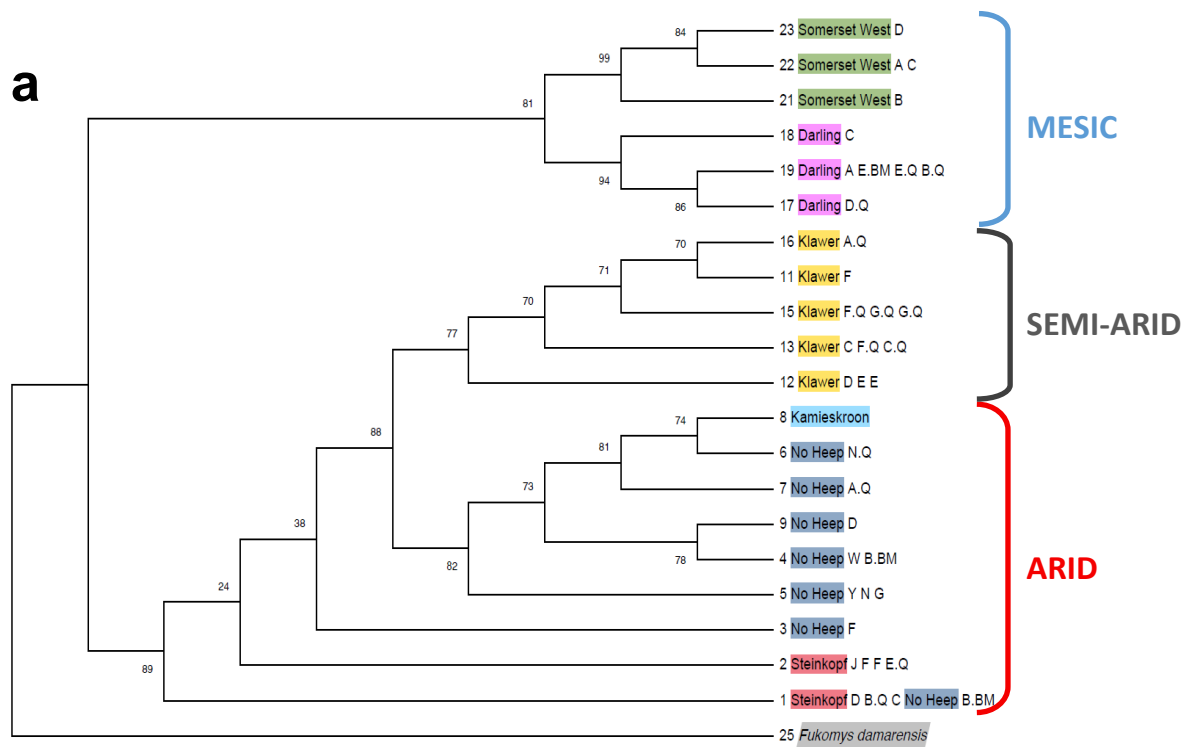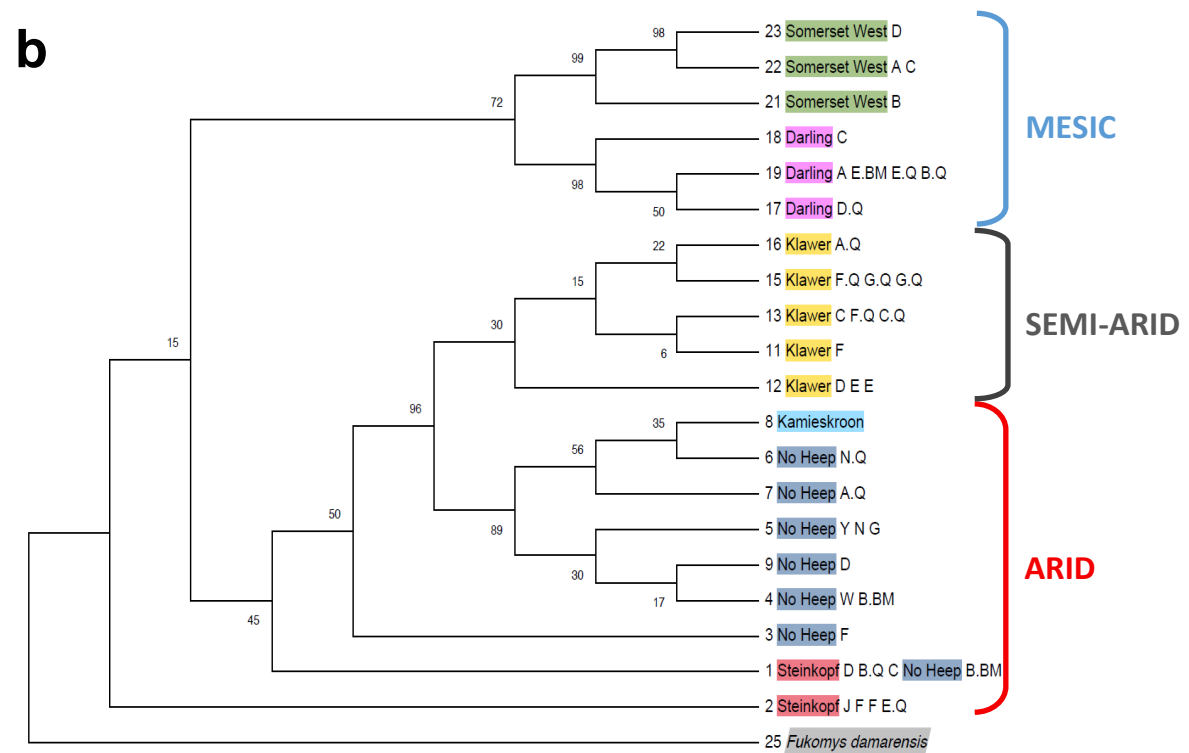

Supplement: evae144_Supplementary_Data [file evae144_supplementary_data.zip › Figure_S1.pdf]

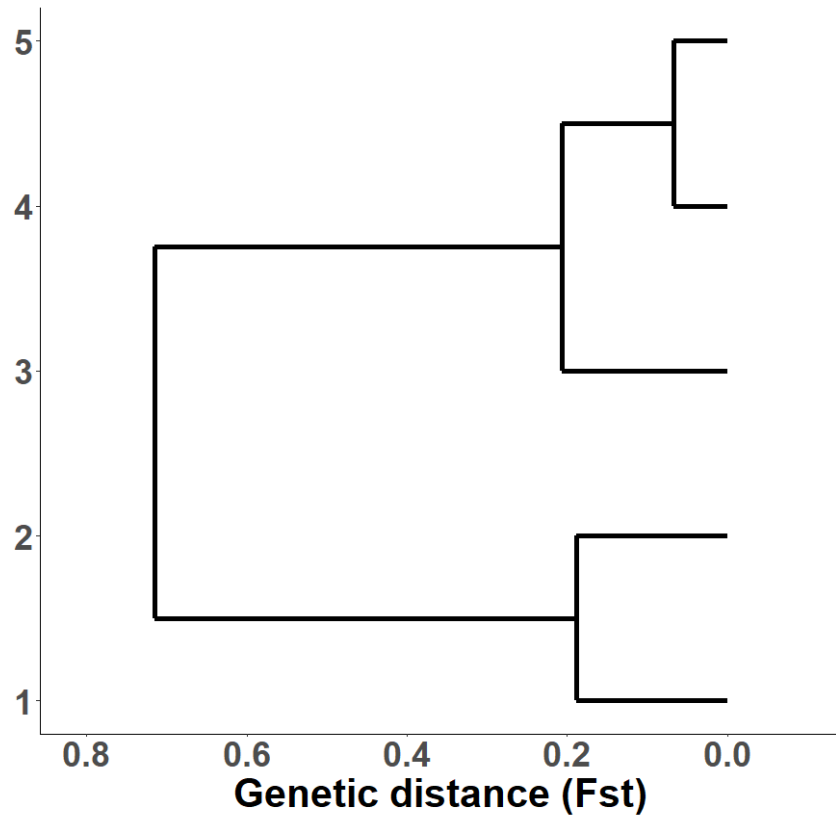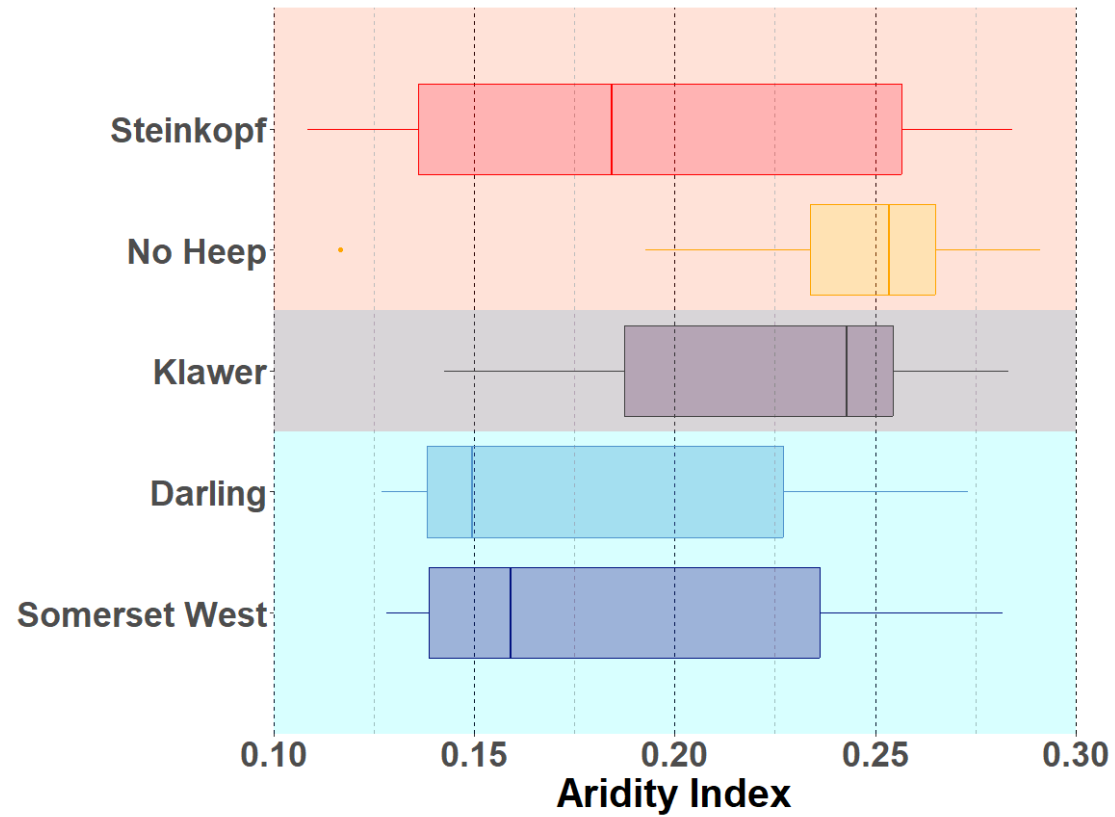

Supplement: evae144_Supplementary_Data [file evae144_supplementary_data.zip › Figure_S2.pdf]

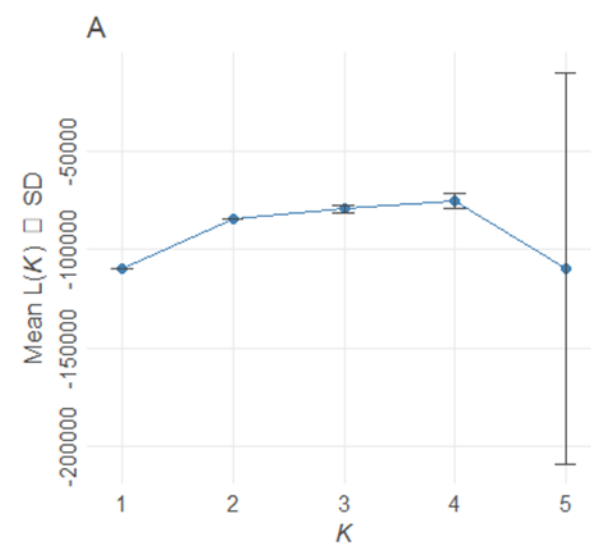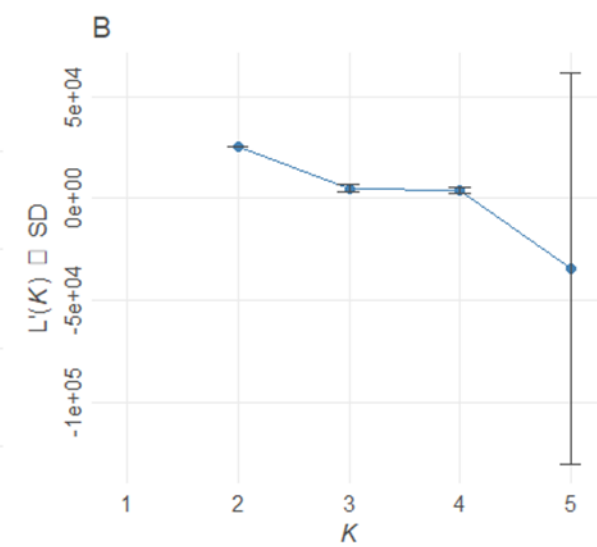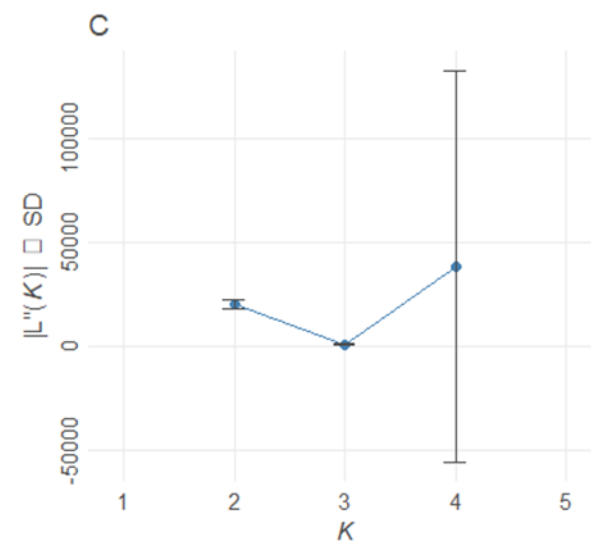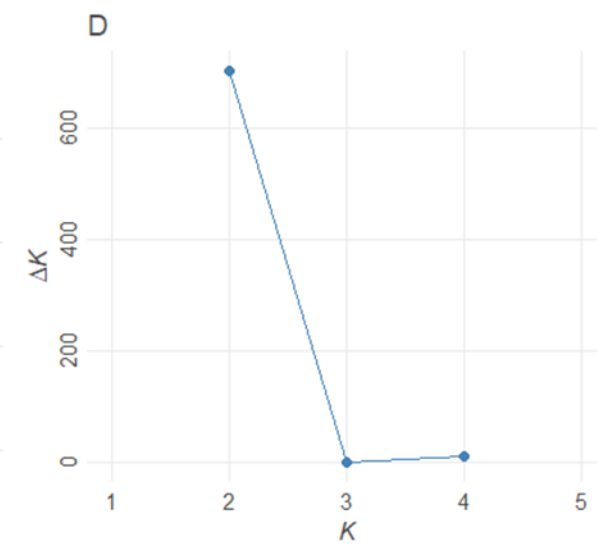

Supplement: evae144_Supplementary_Data [file evae144_supplementary_data.zip › Figure_S3.pdf]

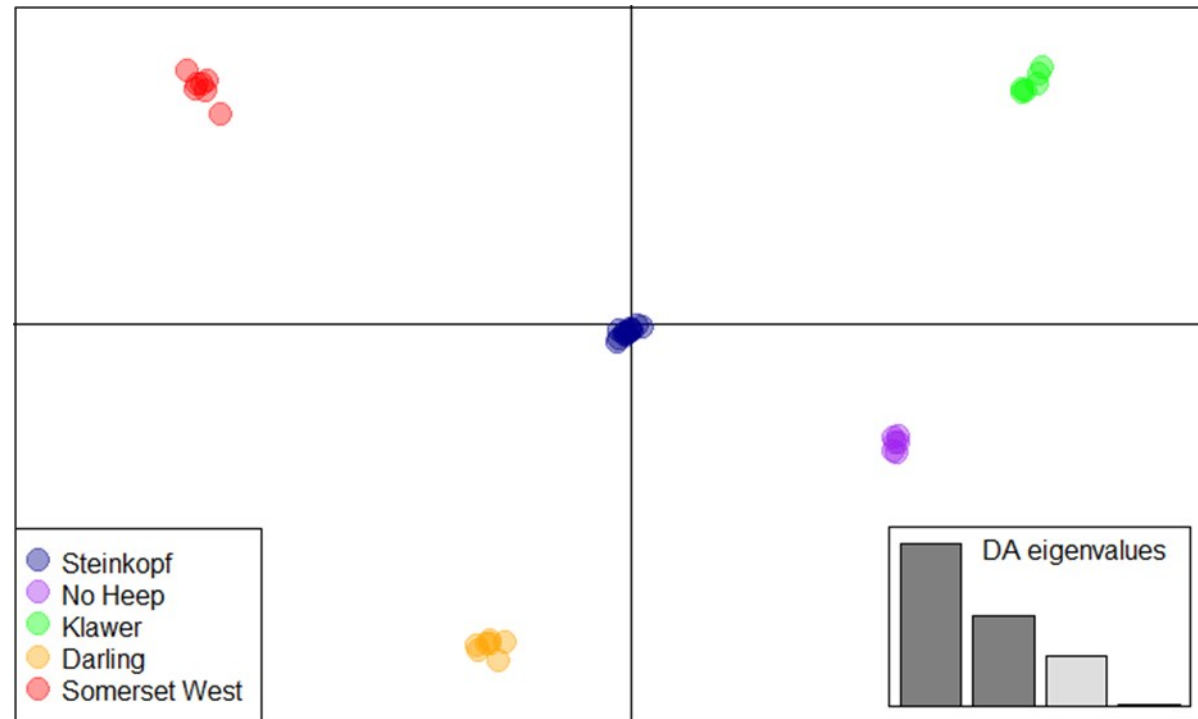

Supplement: evae144_Supplementary_Data [file evae144_supplementary_data.zip › Figure_S4.pdf]
